# Impact of platinum/pemetrexed combination versus other platinum-based regimens on adjuvant chemotherapy in resected lung adenocarcinoma

**DOI:** 10.1038/s41598-017-01347-6

**Published:** 2017-05-03

**Authors:** Xiaoyu Zhai, Qiwen Zheng, Lu Yang, Yixiang Zhu, Junling Li, Yutao Liu, Ziping Wang

**Affiliations:** 1 Department of Medical Oncology, National Cancer Centre/Cancer Hospital, Chinese Academy of Medical Sciences and Peking Union Medical College, Beijing, 100021 China; 2 Medical Insurance Office, Peking University Cancer Hospital & Institute, Beijing, China; 3 Department of Thoracic Medical Oncology, Peking University School of Oncology, Beijing Cancer Hospital & Institute, Beijing, China

## Abstract

For advanced non-squamous non-small cell lung cancer (NSCLC), although platinum/pemetrexed is known to result in a longer survival compared with other regimens, the outcome in the adjuvant setting is still unknown. In this study, the difference of the disease-free survival (DFS) between lung adenocarcinoma patients treated with platinum/pemetrexed and with other platinum-based doublets was concerned. A total of 389 radically resected lung adenocarcinoma patients received adjuvant chemotherapy with platinum/pemetrexed chemotherapy (Group A, n = 143) or other third generation platinum-based regimens (Group B, n = 246) were analyzed in terms of DFS. Propensity score matching (PSM) allowed generation of best matched pairs for the two categories. DFS was proved to be considerably better in pemetrexed doublets group (P = 0.0079); and platinum/pemetrexed was found to be associated with lower rates of several hematological and non-hematological adverse events (AEs), when compared with gemcitabine containing chemotherapy (leukopenia: RR 0.514, p = 0.001; neutropenia: RR 0.688, p = 0.002), or taxanes-doublets treatment (leukopenia: RR 0.685, p = 0.019; neutropenia: RR 0.805, p = 0.032). For patients with radically resected pulmonary adenocarcinoma, adjuvant chemotherapy with platinum/pemetrexed results in a better DFS and a less clinical toxicity in comparison with non-pemetrexed based doublets.

## Introduction

Lung cancer, and non-small cell lung cancer (NSCLC) in particular, is a common malignancy and the leading cause of cancer-related death worldwide^1^. Despite optimal surgical resection for localized NSCLC, the 5-year survival rate without additional treatment is 73% for stage IA disease but declines to 25% for stage IIIA disease. According to the results of several large randomized controlled trials, platinum-based adjuvant chemotherapy (AC) has improved the survival of NSCLC patients with curative resection^2–5^. The Lung Adjuvant Cisplatin Evaluation (LACE) meta-analysis reviewed data from 5 large adjuvant trials of cisplatin-based chemotherapy in NSCLC. The results confirmed the significant effect of postoperative cisplatin-based treatment with both a 5.4% benefit of 5-year overall survival (OS) and a 5.8% benefit of DFS^6^.

Pemetrexed is an antineoplastic agent, which inhibits folate-dependent metabolic processes indispensable to cell replication^7^. Several trials on advanced NSCLC had found that, with a satisfying safety profile, pemetrexed combined with cisplatin showed a promising efficacy comparable with other platinum-based therapy^8, 9^. Platinum/pemetrexed now is recommended as the first-line and maintenance therapy for locally advanced or metastatic non-squamous NSCLC and a single agent pemetrexed regimen is indicated as a second-line therapy^10^. Furthermore, the result of the TREAT study, a phase II trial on early-stage NSCLC, indicated that cisplatin/pemetrexed provokes less toxicity and maintains better dose delivery than cisplatin/vinorelbine; on the other hand, relapse-free survival (RFS) and OS were not influenced by treatment arm^11, 12^.

To date, there exists no published data comparing pemetrexed with other third-generation cytotoxic agents, including paclitaxel, docetaxel and gemcitabine, with regard to clinical toxicity and survival in the adjuvant treatment setting for early-stage lung adenocarcinoma. To address this important gap, we undertook a retrospective study to assess the association between clinical toxicity and various platinum-based doublets and to evaluate the survival associated with these therapy regimens.

## Materials and Methods

### Patients

**Figure 1:**
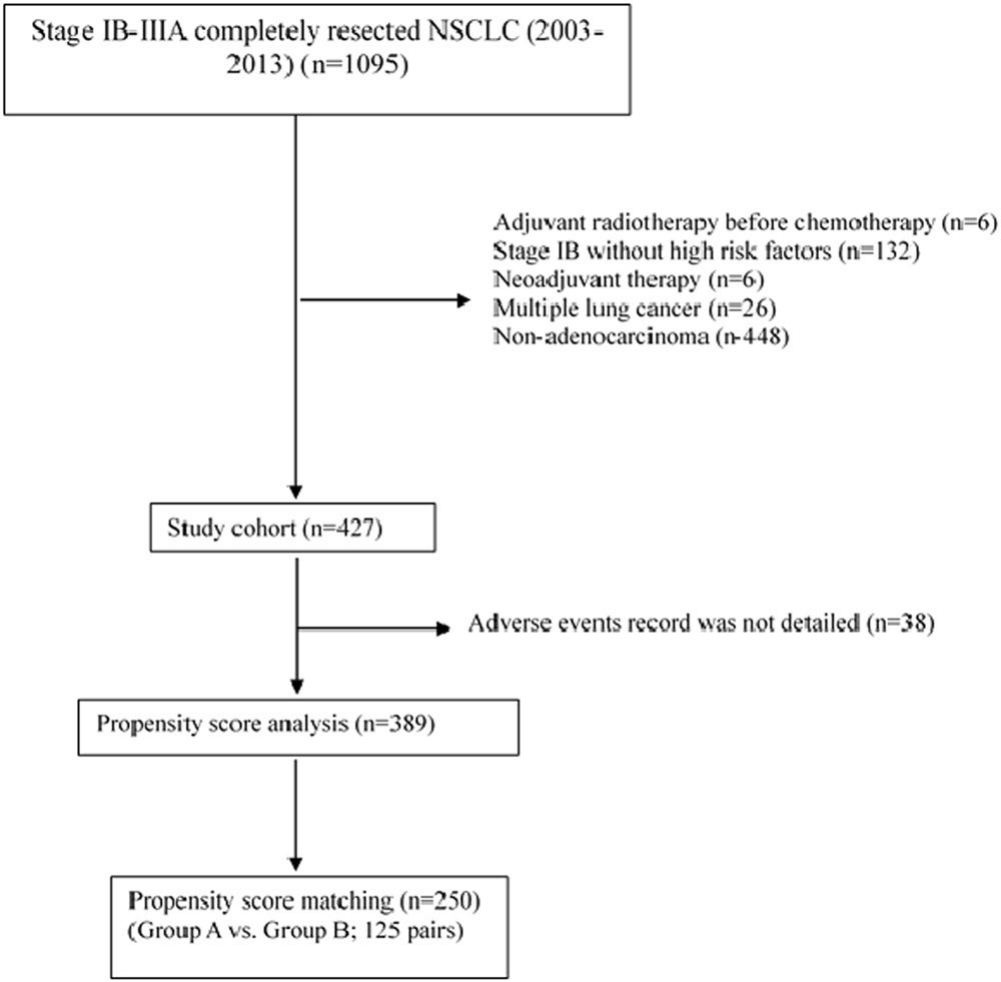
Flow Chart for Selection of Patients.

This retrospective study included 389 consecutive patients who underwent curative resection of lung adenocarcinoma in the Cancer Hospital of the Chinese Academy of Medical Sciences (Beijing, China) between January 2003 and December 2013 (Fig. 1). Inclusion criteria were patients who had fully recovered after resection of pathologically confirmed NSCLC stages (according to the *TNM Classification of Malignant Tumors* (*7th Edition*)^13^) IB, IIA, IIB or IIIA and received postoperative AC, and eligible tumor type was adenocarcinoma. Exclusion criteria were based on histologic type other than adenocarcinoma, prior neoadjuvant chemotherapy, stage IB disease without high-risk factors in terms of poorly differentiated tumors, vascular invasion, wedge resection, tumors >4 cm, visceral pleural involvement, and incomplete lymph node sampling [Nx]. Patients with stage IA, IIIB, or IV were also not eligible. Patients were classified on the basis of age at diagnosis, gender, smoking history, tumor differentiation, pathologic stage, type of resection, lymphatic involvement stage, use of adjuvant radiotherapy, Eastern Cooperative Oncology Group (ECOG) performance status, comorbidity score and number of chemotherapy cycles. All patients who underwent pulmonary resection were followed up from the day of surgery.

### Comorbidity and outcomes

Based on all non-cancer diagnosis records in the hospital files before surgery, comorbid disorders were assessed by Charlson Comorbidity Index (CCI)^14–16^. The primary endpoint of the study was DFS, which is defined by the time from surgery to recurrence (local, regional, and/or distant) or death from any cause. Data was censored on the last contact date. Patients who were still alive at the final follow-up (December 20, 2014) were regarded as censored, and the duration between the initial operation and the final follow-up was included in the survival analysis. The secondary endpoint was to evaluate clinical toxicity in all enrolled patients on an intention-to-treat basis. The *National Cancer Institute* (*NCI*) *Common Terminology Criteria for Adverse Events* (*CTCAE*) *version 3.0* was used to grade toxicities^17^. The identified AEs related with the platinum-based regimens were classified as hematological or non-hematological. Both the hematological AEs noted in medical records and obtained from laboratory examination of blood during follow-up were used to decide the extent of chemotherapy-associated hematological AE in order to minimize the risk of losing information. The estimation of non-hematological AEs was only based on medical records.

### Statistical analysis

All statistical analysis was performed by using *SAS 9.3 software* (SAS Institute Inc., Cary, North Carolina, U.S.A.). Baseline characteristics were presented by applying descriptive statistics. A chi-square test was utilized to compare categorical data. Survival curves were generated by using Kaplan-Meier methods. *GraphPad Prism 5.0* was used to present the survival curves. Univariate analyses were performed by using the log-rank test and multivariable analysis by using Cox proportional hazard regression model. All statistical tests were two-tailed with p < 0.05 set as significant. To reduce the influence of potential confounding factors and to generate comparable study arms, propensity score matching (PSM) method was applied. Variables were gender, age, smoking history, tumor differentiation, pathological stage, use of adjuvant radiotherapy, ECOG PS, comorbidity score, type of resection and number of chemotherapy cycles. After greedy matching, patients with an equivalent propensity score in the two groups were selected by 1:1 matching without replacement. Subgroup analyses were conducted by Cox regression method to investigate whether any significant difference exists between different ages, gender, smoking history, differentiation, pathological stage, type of resection, PS, comorbidity score, adjuvant radiotherapy and number of chemotherapy cycles.

## Results

### Patient characteristics

The mean age of the patients was 56 (range 25–76) years, and the proportion of male and female patients was 50.6% *vs*. 49.4%. Characteristics of the 389 patients are shown in Table 1. The baseline characteristics with significant difference included performance status, use of adjuvant radiotherapy and number of chemotherapy cycles. Patients included in the analysis were then classified into two groups: Group A (*n* = 143), comprising the patients that received adjuvant chemotherapy with platinum/pemetrexed regimen, and Group B (*n* = 246), comprising the patients that received non-pemetrexed platinum-based regimens (146 patients received platinum/paclitaxel, 8 patients platinum/docetaxel, 63 patients platinum/gemcitabine, and 29 patients platinum/vinorelbine). Comparing the percentages of cisplatin and carboplatin use, cisplatin predominated in Group A patients (70%), whereas carboplatin was administered with almost equal frequency to patients in Group B (48.8%).

**Table 1:** Patients demographic characteristics and propensity score-matched characteristics between two groups.

Characteristic	Before PSM			After PSM		
	Pemetrexed (n = 143)	Non- Pemetrexed (n = 246)	P value	Pemetrexed (n = 125)	Non- Pemetrexed (n = 125)	P value
Age						
≤65	124	206	0.467	109	107	0.854
>65	19	40		16	18	
Gender						
Male	73	124	0.917	63	64	1.0
Female	70	122		62	61	
Smoking history						
No	87	153	0.829	73	77	0.699
Yes	56	93		52	48	
Differentiation						
Poorly	28	55	0.475	26	27	0.750
Moderately	102	171		93	88	
Well	9	19		5	7	
Unknown	4	1		1	3	
Pathologic Stage						
IB	21	37	0.178	21	20	0.536
IIA	27	69		33	26	
IIB	9	10		5	9	
IIIA	86	130		66	70	
Type of Resection						
Lobectomy	138	235	0.894	123	120	0.399
Pneumonectomy	4	9		1	4	
Wedge resection	1	2		1	1	
Performance status						
0	48	43	<0.001	31	32	1.0
1	95	202		94	93	
2	0	1		0	0	
Charlson						
0	103	172	0.349	89	90	0.932
1	25	57		27	21	
2	12	15		8	11	
3	3	2		1	3	
Adjuvant radiotherapy						
No	103	218	<0.001	101	101	1.0
Yes	40	28		24	24	
Cycle						
<4	4	24	0.027	5	3	0.732
=4	134	210		115	118	
>4	5	12		5	4	

After adjustment of propensity score matching and variables of gender, age, histologic subtype, smoking history, tumor differentiation, pathological stage, use of adjuvant radiotherapy, performance status and comorbidity score, type of resection and number of chemotherapy cycles, the two groups were well-matched (125 patients each) without significant differences in baseline characteristics (Table 1). The summary shows that the most often used platinum combined with pemetrexed was cisplatin (69.6%) and in the other group it was carboplatin (56.8%).

### Survival Outcome

**Figure 2:**
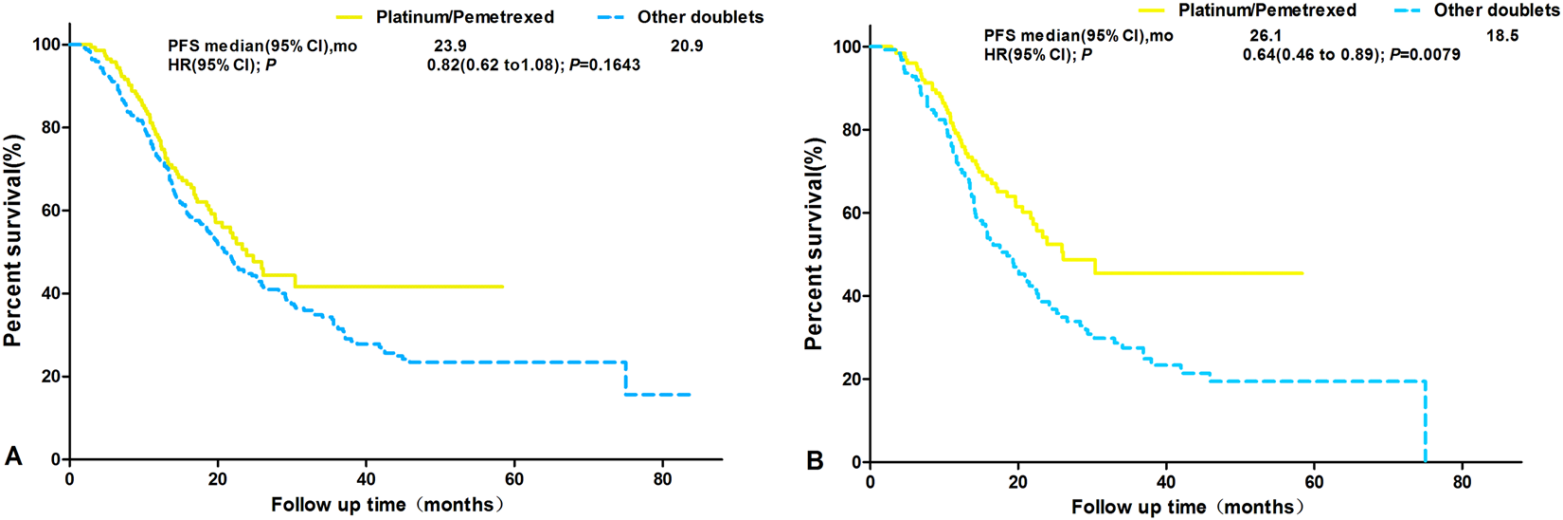
Kaplan-Meier Curves of DFS before (**A**) and after (**B**) PSM.

The results of analysis with Cox proportional hazard regression model before matching are shown in Table 2. Multivariate analysis showed that the tumor histology, pathologic stage, PS, use of adjuvant radiotherapy and number of chemotherapy cycles had significant impact on patient survival, while the use of pemetrexed did not turn out to be an independent prognostic factor of DFS (HR = 0.759, 95%CI: 0.563–1.023, p = 0.07). DFS was not significantly different between the two groups before propensity score-matched (*P* = 0.16) (Fig. 2A). However, after PSM, patients who received adjuvant chemotherapy with platinum/pemetrexed regimen show better DFS than those who did not (*P* = 0.0079) (Fig. 2B).

**Table 2:** Multivariate Cox proportional hazards model of disease-free survival.

Variable		HR	95% CI	P
Pathologic stage	IIA vs IB	2.718	1.498–4.930	0.001
	IIB vs IB	1.976	0.823–4.744	0.128
	IIIA vs IB	6.109	3.480–10.723	<0.001
Performance status	≥1 vs 0	0.62	0.445–0.864	0.005
Adjuvant radiotherapy	Yes vs No	0.684	0.481–0.972	0.034
Cycle	=4 vs < 4	1.337	0.799–2.238	0.268
	>4 vs < 4	2.477	1.208–5.082	0.013
Platinum/pemetrexed	Pem vs Other	0.759	0.563–1.023	0.07

### Exploratory subgroup analysis

**Figure 3:**
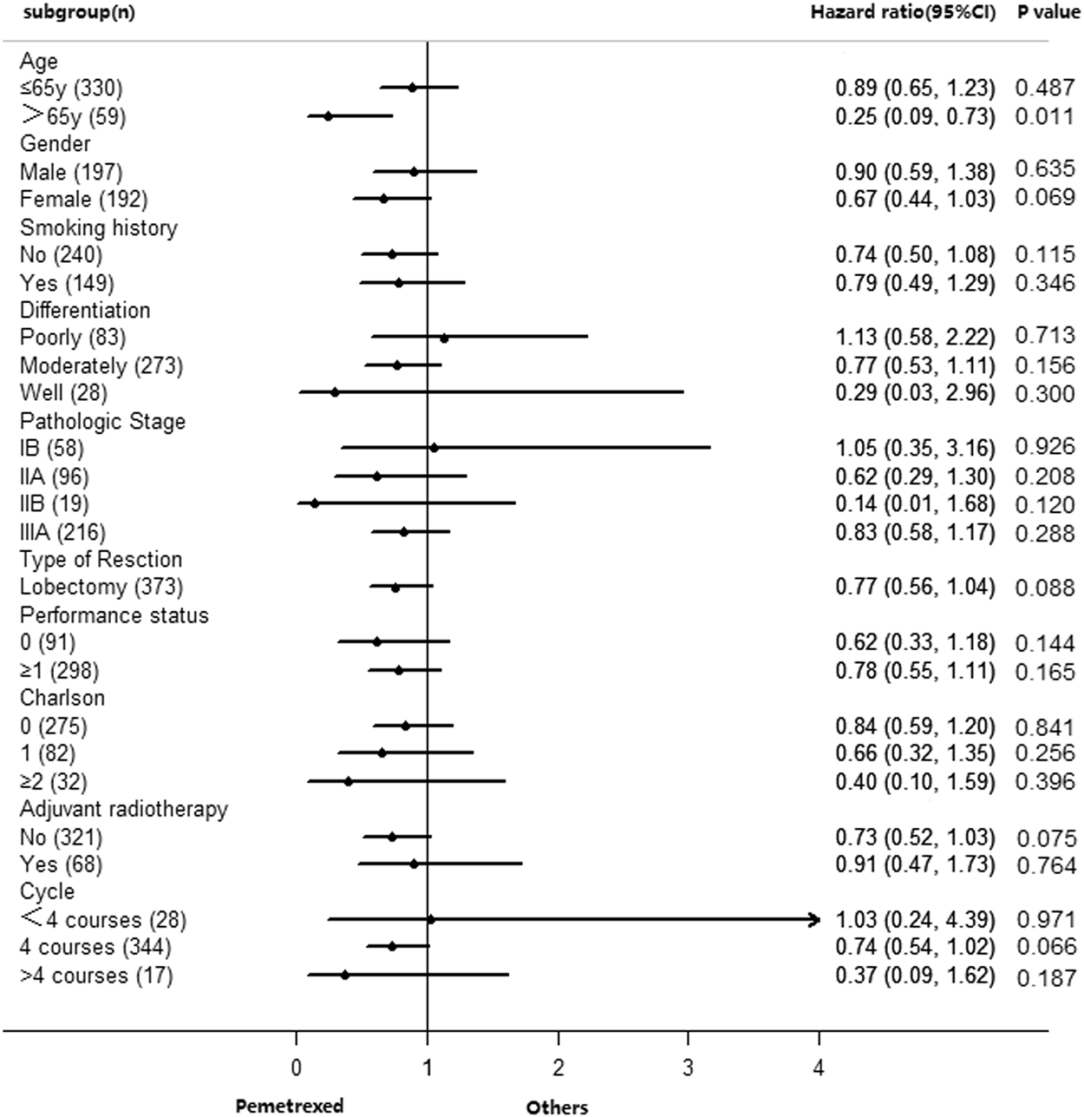
Subgroup Analysis.

Exploratory subgroup analysis was performed to see whether the use of pemetrexed had any significant impact on survival independent of age, gender, smoking history, histology subtype, tumor differentiation, lymphatic involvement stage, pathological stage, use of adjuvant radiotherapy, performance status, comorbidity score, type of resection or number of chemotherapy cycles. From the analysis, pemetrexed benefit is consistent across different subgroups, and especially age >65 years was associated with the decision to use platinum/pemetrexed (HR = 0.25, 95%CI 0.09–0.73, P = 0.011) (Fig. 3).

### Clinical toxicity

Propensity score methods created 107 best-matched pairs for platinum/pemetrexed versus platinum/paclitaxel and platinum/docetaxel (due to the small case number of docetaxel, we combined the 2 regimen), 56 best-matched pairs for platinum/pemetrexed versus platinum/gemcitabine, and 24 best-matched pairs for platinum/pemetrexed versus platinum/vinorelbine for comparisons of clinical toxicity. With adjusting bias after propensity score analysis, platinum/pemetrexed was confirmed to have a significantly lower hematological toxicity than gemcitabine (leukopenia: RR 0.514, p = 0.001; neutropenia: RR 0.688, p = 0.002) as well as paclitaxel- and docetaxel-based chemotherapy (leukopenia: RR 0.685, p = 0.019; neutropenia: RR 0.805, p = 0.032). Comparisons of non-hematological AEs between PSM groups indicated that platinum/pemetrexed treatment was associated with less alopecia than the combination of platinum with docetaxel or paclitaxel (10.28% vs. 20.56%, RR 0.500, p = 0.037). Platinum/pemetrexed was also associated in fewer patients with constipation than the platinum/gemcitabine doublet (0% vs. 25%, p = 0.022) (Table 3). Moreover, both grade 3/4 hematologic toxicity and non-hematologic toxicity were significantly lower for platinum/pemetrexed compared with other platinum-based doublets (Table 4). Otherwise, platinum/pemetrexed was associated with more vomiting when compared with paclitaxel- and docetaxel-based doublet (52.34% vs. 22.43%, RR 2.333, p < 0.001). All other toxicities were not significantly different. (Table 3), and a similar result was found in the 125 well-matched pairs (Table 5).

**Table 3:** Comparing incidences of hematological and non-hematological AEs between the propensity score matched treatment group.

Treatment Comparison	Platinum/Pemetrexed vs. Platinum/Gemcitabine				RR	P *	Platinum/Pemetrexed vs. Platinum/Vinorelbine				RR	P *	Platinum/Pemetrexed vs. Platinum/Paclitaxel+Docetaxel				RR	P*
Matched pairs	56 pairs						24 pairs						107 pairs					
All grades AE	N	%	N	%			N	%	N	%			N	%	N	%		
**Non-hematological**																		
Fatigue	10	17.86%	11	19.64%	0.909	0.809	6	25.00%	8	33.33%	0.750	0.525	17	15.89%	19	17.76%	0.895	0.715
Fever	0	0.00%	2	3.57%	—	0.495	0	0.00%	1	4.17%	—	1.000	0	0.00%	3	2.80%	0.000	0.246
Nausea	47	83.93%	52	92.86%	0.904	0.140	21	87.50%	22	91.67%	0.955	0.637	90	84.11%	82	76.64%	1.098	0.169
Vomiting	30	53.57%	28	50.00%	1.071	0.705	13	54.17%	10	41.67%	1.300	0.386	56	52.34%	24	22.43%	2.333	**<0.001**
Mucositis	1	1.79%	2	3.57%	0.500	1.000	1	4.17%	1	4.17%	1.000	1.000	1	0.93%	2	1.87%	0.500	1.000
Constipation	2	3.57%	4	7.14%	0.500	0.679	0	0.00%	6	25.00%	—	**0.022**	3	2.80%	2	1.87%	1.500	1.000
Rash	1	1.79%	1	1.79%	1.000	1.000	1	4.17%	0	0.00%	—	1.000	4	3.74%	0	0.00%	—	0.121
Alopecia	9	16.07%	5	8.93%	1.800	0.253	1	4.17%	4	16.67%	0.250	0.348	11	10.28%	22	20.56%	0.500	**0.037**
**Hematological**																		
Leukopenia	18	32.14%	35	62.50%	0.514	**0.001**	8	33.33%	11	45.83%	0.727	0.376	37	34.58%	54	50.47%	0.685	**0.019**
Neutropenia	33	58.93%	48	85.71%	0.688	**0.002**	15	62.50%	19	79.17%	0.789	0.204	62	57.94%	77	71.96%	0.805	**0.032**
Anemia	22	39.29%	28	50.00%	0.786	0.254	10	41.67%	11	45.83%	0.909	0.771	40	37.38%	49	45.79%	0.816	0.212
Thrombocytopenia	14	25.00%	15	26.79%	0.933	0.829	4	16.67%	7	29.17%	0.571	0.303	21	19.63%	20	18.69%	1.050	0.862

AE, adverse event; RR, rate ratio. *P values less than 0.05 were in bold to indicate significant differences.

**Table 4:** Toxic effects.

Toxicity	Platinum/Pemetrexed	Platinum/Gemcitabine	Platinum/Pemetrexed	Platinum/Vinorelbine	Platinum/Pemetrexed	Platinum/Paclitaxel or Docetaxel
	N = 56		N = 24		N = 107	
Hematologic toxicity G3/4 (%)	32.1	73.2	50	58.3	51.4	72.9
WBC	8.9	19.6	20.8	20.8	25.2	29.0
Neutropenia	23.2	44.6	29.2	33.3	26.2	40.2
Anemia	0	3.6	0	0	0	0
Thrombocytopenia	0	5.4	0	4.2	0.9	3.7
Non-hematologic toxicity G3/4 (%)	0	19.6	0	16.7	2.7	8.4
Fatigue	0	0	0	0	0	0
Fever	0	0	0	0	0	0
Nausea	0	10.7	0	8.3	0.9	4.7
Vomiting	0	8.9	0	4.2	0.9	0.9
Mucositis	0	0	0	0	0	0
Constipation	0	0	0	0	0	0
Rash	0	0	0	0	0	0
Alopecia	0	0	0	4.2	0.9	2.8

**Table 5:** Comparing incidences of toxic effects between the propensity score matched treatment group.

		Pemetrexed		Non- Pemetrexed		P
		125 pairs				
Adverse events		N	%	N	%	
Fatigue	AE	18	14.29%	26	20.63%	0.184
	SAE	0	0	0	0	—
Fever	AE	1	0.79%	3	2.38%	0.622
	SAE	0	0	0	0	—
Nausea	AE	102	81.60%	99	79.20%	0.633
	SAE	1	0.79%	6	4.76%	0.12
Vomiting	AE	64	50.79%	34	26.98%	<0.001
	SAE	0	0	4	3.17%	0.122
Mucositis	AE	2	1.59%	4	3.17%	0.684
	SAE	0	0	0	0	—
Constipation	AE	8	6.35%	5	3.97%	0.393
	SAE	0	0	0	0	—
Rash	AE	4	3.17%	0	0.00%	0.122
	SAE	0	0	0	0	—
Alopecia	AE	7	5.56%	29	23.20%	<0.001
	SAE	0	0	3	2.38%	0.247
WBC	AE	57	45.60%	98	78.40%	<0.001
	SAE	4	3.17%	28	22.22%	<0.001
Neutropenia	AE	69	55.20%	95	76.00%	0.001
	SAE	14	11.11%	56	44.44%	<0.001
Anemia	AE	44	35.20%	57	45.24%	0.124
	SAE	0	0	0	0	—
Thrombocytopenia	AE	21	16.67%	24	19.05%	0.622
	SAE	3	2.38%	3	2.38%	1

AE, adverse event SAE, sever adverse event.

## Discussion

Patients recruited for clinical trials according to strict inclusion criteria may not inevitably match with unselected patient populations. Compared with general populations, they are usually younger and present with better general conditions and fewer co-existing diseases, thus not representing typical characteristics of patients from daily clinical routine. Well-designed retrospective studies, therefore, can provide important ancillary information for the “real world situation”^18^.

To the best of our knowledge, our analysis is the first “real-world” study comparing survival results and clinical toxicity associated with pemetrexed/platinum-based doublets and other third-generation doublets routinely used in the adjuvant chemotherapy for completely resected lung adenocarcinoma. Pemetrexed/platinum doublets were shown to be less hematotoxic, particularly regarding leukopenia and neutropenia, in this way increasing the safety of adjuvant chemotherapy for NSCLC. Though we noticed that pemetrexed/platinum treatment was associated with more vomiting than the other combinations. This observation most probably finds its explanation in the higher proportion of cisplatin used instead of carboplatin as combination partner for pemetrexed compared with the other regimens, because cisplatin is known to be one of the most emetogenic cytostatic. The risk of vomiting associated with cisplatin is greater than with non-cisplatin-containing regimens^19, 20^.

In our study, we utilized propensity score matched methods (PSM) to compare survival data and clinical toxicity. PSM analysis allowed us to compare survival between patients with similar background characteristics^21^. When potentially confounding variables (gender, age, smoking history, tumor differentiation, pathological stage, use of adjuvant radiotherapy, ECOG PS, comorbidity score, type of resection and number of chemotherapy cycles) were adjusted, patients who received a doublet of platinum/pemetrexed had significantly better DFS than those treated with other third-generation platinum-based doublets. This strongly indicates that adjuvant therapy with platinum/pemetrexed doublets improves survival of patients with completely resected lung adenocarcinoma. The indication generated in our study should have profound significance for clinical research and future studies.

Although a phase II trial showed that adjuvant chemotherapy with cisplatin/pemetrexed yielded less toxicity and better dose delivery than vinorelbine combined with cisplatin, DFS was not influenced by chemotherapy type^11, 12, 22^. The superior survival data related with pemetrexed observed in our study may be the result of a potential effect of tumor histology. The primary mechanism of action of pemetrexed is the inhibition of the enzyme thymidylate synthase (TS). Docetaxel, paclitaxel, as well as vinorelbine can destroy the mitotic activity of tumor cells^23^, and gemcitabine is a nucleoside analog^24^. The anti-cancer activities of the last four cytotoxic drugs, directed to cell replication, are less sensitive to tumor histology^25^. As a biomarker, TS expression is potentially associated with the response to pemetrexed-based chemotherapy in NSCLC patients^26, 27^ and had become an important determinant of survival for stage I NSCLC^28^. Moreover, the differential efficacy of pemetrexed in advanced NSCLC on the basis of tumor histology has been reported in a phase III study^8^. The better survival and clinical efficacy achieved with pemetrexed/platinum doublets in this study might be explained by the restriction to adenocarcinoma histology, because adenocarcinomas express TS, a key enzymes inhibited by pemetrexed^29^. We also found that the survival associated with platinum/pemetrexed in our study was better than that reported in the randomized phase II TREAT study^11, 12^. Reported relapse-free survival did not differ between cisplatin/pemetrexed and cisplatin/vinorelbine in the TREAT study. The different outcome between our study and TREAT study is probably caused by a disparity in patient pathological types. The tumor histology in our study was uniformly adenocarcinoma, which shows lower TS expression, a confirmed predictive marker for better response to pemetrexed-based chemotherapy^27, 30^. Eventually, the better survival and reduced clinical toxicity observed in our study with pemetrexed-based adjuvant therapy need further evaluation with a better research design to confirm a potential effect on overall survival in lung adenocarcinoma.

The LACE meta-analysis and a retrospective analysis of the JBR.10 trial consistently reported that adjuvant chemotherapy significantly improved survival, and that treatment-related mortality did not differ by age^31, 32^. Thus, the role of adjuvant chemotherapy should not be underestimated in elderly patients. In this study, in patients older than 65 years, the use of platinum/pemetrexed resulted in better DFS than other doublets. By contrast, there is no overall difference in DFS between patients who received platinum/pemetrexed and other third-generation doublets. In the subgroups stratified by gender, smoking history, tumor differentiation, pathological stage, use of adjuvant radiotherapy, ECOG PS, comorbidity score, type of resection and number of chemotherapy cycles, we also could not identify patients who benefited more from pemetrexed. Thus, patients’ age is a prognostic factor for DFS in patients with completely resected NSCLC.

This is the first study to compare the impact on survival of different regimens for completely resected NSCLC; however, it also has some limitations. First, our study was limited by retrospective nature of the analysis. Although we performed multivariate analysis and used PSM method to eliminate the selection bias as much as possible, some disparities of both known and unknown prognostic factors, such as total dose of chemotherapy, may affect the results. Second, the exploration of OS in this study was limited. As we know, there are different therapies for regional recurrences or distant metastases, governed by different pathological or mutational types. Several studies^33, 34^ indicated that in advanced NSCLC patients with EGFR mutations, a first-line therapy with gefitinib resulted in encouraging clinical therapeutic outcomes. Furthermore, the use of pemetrexed plus cisplatin showed significant benefits with regard to survival in advanced-stage NSCLC patients with adenocarcinoma and large-cell carcinoma^8^. Knowing that several factors can affect the OS outcome, we set DFS as the primary end point of this study. Third, because of the relatively small sample size of other platinum-based doublets, this study could not compare the effect on survival among each chemotherapy regimens. In addition, because the histological type of NSCLC of all patients in our study is adenocarcinoma, it is still unclear whether there is a correlation of histology with outcome of adjuvant chemotherapy. The predictive efficacy of pemetrexed activity according to the tumor histology was reported, in a phase III trial, only in patients with advanced NSCLC^8^. However, so far no prospective trial or meta-analysis in early-stage NSCLC has reported the relation between histology and outcomes. In JBR.10, squamous histologic features (P = 0.002) were associated with significantly prolonged recurrence-free survival. Though cisplatin/vinorelbine had a positive impact in the ANITA trial, a poor outcome in adenocarcinoma was also reported^3, 6, 35–38^. Likewise, histological type had no impact on adjuvant treatment in LACE study subgroup analysis of vinorelbine^39^. Accordingly, in adjuvant therapy of early-stage NSCLC, the predictive effect of tumor histology still need further evaluation.

## Declaration

### Ethics approval and consent to participate

The study protocol was approved by the institutional review board at the Cancer Hospital, Chinese Academy of Medical Sciences, and PUMC in accordance with the Declaration of Helsinki. Patients provide informed consent authorizing the use of their personal information for research purposes.
